# Safety Evaluation of Chemical Insecticides to *Tetrastichus howardi* (Hymenoptera: Eulophidae), a Pupal Parasitoid of *Spodoptera frugiperda* (Lepidoptera: Noctuidae) Using Three Exposure Routes

**DOI:** 10.3390/insects13050443

**Published:** 2022-05-07

**Authors:** Zhuo Liu, Hao Su, Baoqian Lyu, Sanqiang Yan, Hui Lu, Jihong Tang

**Affiliations:** 1 State Key Laboratory Breeding Base of Green Pesticide and Agricultural Bioengineering, Key Laboratory of Green Pesticide and Agricultural Bioengineering, Ministry of Education, Guizhou University, Guiyang 550025, China; yxzhuoliu@163.com; 2 Key Laboratory of Integrated Pest Management on Tropical Crops of Ministry of Agriculture and Rural Affairs, Environment and Plant Protection Institute, Chinese Academy of Tropical Agricultural Sciences, Haikou 571101, China; qtsuhao@163.com (H.S.); yansanq@163.com (S.Y.); aaaluhui@163.com (H.L.); jihong_23@163.com (J.T.); 3 Hainan Key Laboratory for Biosafety Monitoring and Molecular Breeding in the Nanfan Area, Sanya Research Institute, Chinese Academy of Tropical Agricultural Sciences, Sanya 572025, China; 4 College of Forestry, Hainan University, Haikou 570228, China

**Keywords:** pest, insecticides, pupal parasitoid, toxicity, sublethal effects

## Abstract

**Simple Summary:**

*Spodoptera frugiperda* is an important pest in many crops worldwide, causing substantial economic losses. The main control strategies are biological control and chemical control. However, pesticides also have varying degrees of toxicity to parasitic wasps in the field. In this study, we evaluate the safety of insecticides for *Tetrastichus howardi*, an important pupal parasitoid of *S. frugiperda*. We tested the toxicity of six major control insecticides against the parasitic wasps. Indoxacarb was the less toxic pesticide to *T. howardi* due to its toxicity’s low-risk quotient (7.43). Furthermore, we used three methods to simulate the side effects of different concentrations of indoxacarb on *T. howardi*. The exposure of adults to pesticide residues on the glass tube was the most significant in inhibiting development and fecundity. Moreover, *T*. *howardi* had a lower parasitism rate and emergence rate with the higher pesticide concentrations. Overall, our study showed that different exposure patterns and concentrations of pesticides have varying degrees of side effects on *T. howardi*. Even if the pesticide residues are low, such exposures can affect the life cycle of parasitic wasps, affect their population establishment, and thus affect pest control. This study guides a more scientific and comprehensive pesticide application and releases natural enemies.

**Abstract:**

*Spodoptera frugiperda* has become a major pest in many crops worldwide. The main control strategies are biological and chemical controls. However, pesticides have varying degrees of toxicity to parasitic wasps in the field. To integrate chemical and biological controls, we evaluated the safety of insecticides to *Tetrastichus howardi*, an important pupal parasitoid of *S. frugiperda*. This study assessed the toxicity of six major control insecticides (emamectin benzoate, chlorfenapyr, indoxacarb, chlorantraniliprole, bisultap, and lufenuron) to *T. howardi* based on risk quotient. The results showed that indoxacarb had the lowest risk quotient (RQ = 7.43). Then the side effects of three sublethal concentrations (LC_20_, LC_30_, LC_40_) of indoxacarb were tested using three methods (1. Adult exposure to pesticide residues on the glass tube; 2. Adult exposure to pesticide residues on the host; 3. Larval exposure to pesticides through host exposure). Overall, *T*. *howardi* had a lower parasitism rate and emergence rate with the higher pesticide concentrations. Furthermore, among three methods, the adult exposure to pesticide residues on the glass tube was the most efficient in inhibiting the parasitism rate, and impairing the emergence rate and the offspring female/male ratio. This study guides a more scientific and comprehensive application of pesticides and releases natural enemies in the field.

## 1. Introduction

Integrated pest management (IPM) programs used combination strategies such as biological control and chemical control to manage populations of pests below economically damaging levels [1]. Chemical insecticides have been widely used starting from the last century, and they are the mainstream technology for pest control due to their high efficiency and easy operation. However, chemical control can adversely affect parasitic wasps’ physiological and behavioral, which are important beneficial arthropods in biological control [2,3,4,5]. These effects usually occur days following initial pesticide application [6]. Even if concentrations are low, such exposures may reduce their ability to provide ecosystem services [7]. It is advisable to use pesticides that are effective against target pests but have few adverse effects on non-target organisms. The safety evaluation of chemical insecticides on natural enemies is imperative to coordinate chemical and biological control.

People often pay more attention to the acute toxicity of pesticides on beneficial insects in the past [8]. The application of pesticides in the field directly affects parasitic wasps through spray droplets. It may also have sublethal effects due to pesticide residues. The lethal and sublethal effects of parasitic wasps exposed to pesticides through different ways are also different. Parasitic wasps may turn to the region where insecticide treatments have been carried out from the untreated surrounding. They may parasitize the pests (with residues) after insecticide application, and the larval parasitoids may already be in the host during pesticide treatment [9].

*Tetrastichushowardi* (Olliff) (Hymenoptera: Eulophidae) is a parasitoid that elicits the death of the pupae of Lepidoptera [10]. As a case in point, *Spodoptera frugiperda* (J.E. Smith) (Lepidoptera: Noctuidae), a worldwide invasive pest that originated from Tropical America [11], with a wide range of plant feeding and a high migratory capacity, this species has spread rapidly worldwide, causing substantial economic losses [12,13,14]. The *S. frugiperda* continued to spread and was detected in China in January 2019 [15]. Tang et al. found that *T.* *howardi* has an average 4.5% parasitic rate of the pupae of *S. frugiperda* in the field in the Hainan Province, China [16], and has the characteristics of easy propagation and strong thermal adaptation. The number of wasp eggs could be up to 60 in a host pupa [17]. 

Data are available to study the sublethal effect of various insecticides on *Trichogramma achaeae* (Nagaraja and Nagarkatti) (Hymenoptera: Tricogrammatidae) [18], *Trichogramma* *dendrolimi* (Matsumura) (Hymenoptera: Tricogrammatidae), *Trichogramma ostriniae* (Pang and Chen) (Hymenoptera: Tricogrammatidae), and *Trichogramma* *confusum* (Viggiani) (Hymenoptera: Tricogrammatidae) [19]. However, there is little information about the sublethal effects of pesticides on *T. howardi*. In addition, previous studies have focused on the glass vial method used to simulate the direct impact of pesticides on parasitic wasps in the field without considering other ways in which pesticide residues may act on parasitoids. In the present study, we assess the risk quotient of six insecticides (emamectin benzoate, chlorfenapyr, indoxacarb, chlorantraniliprole, bisultap, and lufenuron) to *T.* *howardi*. After that, the experiments on the sublethal effects of the pesticides exhibiting the lowest risk quotient were conducted on parasitoids. Three routes of exposure were simulated in the laboratory (1. Adult exposure to pesticide residues on the glass tube; 2. Adult exposure to pesticide residues on the host; 3. Larval exposure to pesticides through host exposure) to assess the development and fecundity of organisms. 

## 2. Materials and Methods

### 2.1. Insects

The population of *T. howardi* was initially obtained from *S. frugiperda* pupal samples in maize fields in Danzhou, Hainan, China, and then established in the acclimatized chamber (26 ± 2 °C, 70 ± 10% RH, and 12 L:12 D) of the Environment and Plant Protection Institute, Chinese Academy of Tropical Agricultural Sciences (EPPI, CATAS) in Danzhou, Hainan, China. The pupae were maintained inside glass tubes (15 cm × 1.5 cm), and the emerged *T. howardi* adults were then transferred in a ventilated metal-acrylic cage (40 × 40 × 40 cm) containing hosts (24 h old *S. frugiperda* pupae) whose number corresponds to female wasps. After every 24 h, the pupae were transferred into glass tubes. The hosts and 15% (*w*/*v*) honey droplets were daily provided to the parasitoids until their death. The culture of *T. howardi* has been reared for three generations and has never been in contact with pesticides before being used in the experiments. 

### 2.2. Insecticides and Chemicals

Following the recommendation of the Ministry of Agriculture and Rural Affairs of The People’s Republic of China regarding the use of pesticides for emergency control of *S. frugiperda* [20], the emamectin benzoate (95%, Ningxia Taiyixin Biological Technology Co., Ltd., Helan, China), chlorfenapyr (95%, Jiangsu Zhongqi Technology Co., Ltd., Changzhou, China), indoxacarb (94%, FMC Corporation, Philadelphia, PA, USA), Chlorantraniliprole (95.3%, Shanghai Dupont Agrochemical Co., Ltd., Shanghai, China), bisultap (40%, Jiangsu Tianrong Group Co., Ltd., Changzhou, China), and lufenuron (98%, Jiangxi Oushi Chemical Co., Ltd., Ji’an, China) were used for testing. They all have stomach toxicity and contact toxicity. All pesticides were the original drug and they were dissolved in distilled water containing 1% acetone [21]. 

### 2.3. Risk Assessment of Six Insecticides against T. howardi

The risk quotient (RQ) of each pesticide to *T. howardi* was calculated to test the toxicity based on the formula given by Preetha: RQ = Recommended dose (g a.i ha^−1^)/LC_50_ of *T. howardi* [22]. The risk quotient of less than 50 for a pesticide is categorized as harmless (Category 1), 50–2500 as slightly to moderately toxic (Category 2), and more than 2500 as dangerous (Category 3) [23].

For *T. howardi*, the toxicity of six insecticides was determined using the glass vial method [24] on its newly emerged adults (1 d old, no distinction between male and female). Preliminary experiments were performed starting from the recommended field application rate with a set of decreasing serial dilutions to determine the range of insecticide concentrations [14]. The recommended field rate was obtained from the Electronic Pesticide Manual of ICA, MOA, China (http://www.ny100.cn/ (accessed on 22 October 2021)). Then each insecticide was diluted into five concentration gradients increasing with a geometrical progression and poured into a glass tube (15 cm × 1.5 cm) to establish a concentration–mortality relationship. The concentration ranges were as follows: 0.0391–0.625 mg L^−1^ of emamectin benzoate, 0.15–2.4 mg L^−1^ of chlorophenyl, 1.1719–18.75 mg L^−1^ of indoxacarb, 0.3125–5 mg L^−1^ of chlorantraniliprole, 0.1406–2.25 mg L^−1^ of bisultap, and 0.005–50 mg L^−1^ of lufenuron. The tube was manually rotated on a flat surface, allowing the solution to adhere to the wall of the tube evenly and dry under laboratory conditions (26 ± 2 °C, 70 ± 10% RH, and 12 L:12 D). Sixty newly emerged *T. howardi* were then placed into the test tubes treated with the pesticide solutions at different concentrations. Then, 15% (*w*/*v*) honey droplets were provided to the parasitoids. For the experiments, distilled water containing 1% acetone was used as a control. Each bioassay was repeated three times. The parasitoids’ mortality was counted after 24 h acute toxicity, and the median lethal concentration (LC_50_) values were estimated from the probit analysis.

### 2.4. Sublethal Effects of Indoxacarb on T. howardi

**Figure 1:**
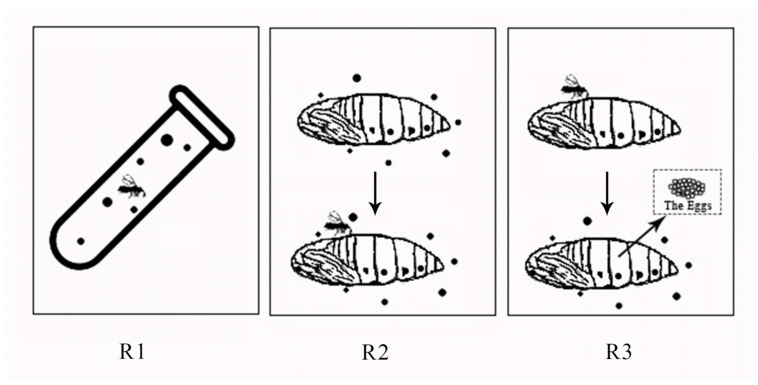
**R1** (Routes of exposure 1) is adult exposure to pesticide residues on the glass tube; **R2** (Routes of exposure 2) is adult exposure to pesticide residues on the host; and **R3** (Routes of exposure 3) is larval exposure to pesticides through host exposure.

Under the previous experiments and based on the regression equation, the LC_20_ (1.72 mg L^−1^), LC_30_ (2.66 mg L^−1^), and LC_40_ (3.75 mg L^−1^) were calculated and used to assess the effect on *T. howardi* development and fecundity, with the control consisting of distilled water containing 1% acetone, for a total of four treatments. Each treatment was repeated three times. Three routes of exposure (Figure 1) were conducted to investigate the sublethal effects. Parasitism rate was the number of parasitized pupae divided by the total number of pupae. Emergence rate was the number of emergence pupae divided by the number of parasitized pupae. The female ratio of offspring was the number of emergence female wasps divided by a total number of emergence wasps. Moreover, the developmental duration was the days from the egg to the adult stage.

Adult exposure to pesticide residues on the glass tube (Routes of exposure 1): In the glass vial method (same as toxicity determination), the newly emerged wasps (within 24 h) were treated with three different sublethal concentrations. After 24 h, 40 pairs of surviving parasitoids were extracted from each treatment group, male and female wasps were placed in a test tube (not treated with pesticides) and allowed to mate. The next day, the pupae of armyworm, which pupated for 1 day, were placed in each test tube and parasitized for 24 h. *T. howardi* was removed and transferred. All the test tubes were fed with cotton balls with 15% honey water and sealed. Whether each pupa emerged, the number of offspring and the female ratio of each parasitic wasp produced were observed and recorded. If eggs, pupae, and larvae of parasitoids were found, they were counted as parasitized successfully. The parasitism rate, emergence rate, and developmental duration were calculated.

Adult exposure to pesticide residues on the host (Routes of exposure 2): For the dipping method [25], the pupae of armyworm pupated for one day were immersed with three different sublethal concentrations of indoxacarb for 15 s and then taken out to air dry. They were placed into 40 test tubes, each containing a pair of *T. howardi* that newly emerged (within 24 h) and mated for 24 h (the conditions and operation were the same as above).

Larval exposure to pesticides through host exposure (Routes of exposure 3): For this dipping method, 40 pairs of newly emerged (within 24 h) *T. howardi* were first placed into the test tube for mating for 24 h. Each pair was put into the test tube with 1-day-old pupae to parasitize for 24 h. The pupae were immersed in the sublethal concentration of indoxacarb for 15 s. The operation and conditions were the same as above. The treatment was repeated three times for every 40 tubes. The statistical period of development, the ratio of offspring to female, the number of emerged offspring, the parasitism rate, and the emergence rate were calculated.

### 2.5. Data Analysis

The LC_20_, LC_30_, LC_40_, LC_50_, and slope were determined using the SPSS. 23 software, based on probit analysis [26]. The risk quotient, and biological parameters, including female ratio, parasitism rate, and developmental duration, underwent statistical analysis using the MS Excel 2010 software. Using ANOVA, followed by Tukey’s multiple range test, all the parameters were tested with the DPS statistical software [27]. The parasitism rate and emergence rate were analyzed using arcsine √ ×-transformed data (*p* < 0.05).

## 3. Results

### 3.1. Risk Assessment of Six Insecticides against T. howardi

Data of toxicity of the insecticides to *T.* *howardi* is summarized in Table 1. Based on LC_50_ values (mg a.i L^−1^), the order of toxicity of the insecticides was as follows: emamectin benzoate (0.09) > chlorfenapyr (0.29) and bisultap (0.30) > chlorantraniliprole (0.84) > lufenuron (0.91) > indoxacarb (5.38) (Table 1). Compared with other chemicals, the RQ of bisultap was significantly higher than that of other pesticides, while the indoxacarb showed the lowest. These chemicals were sorted into two categories based on RQ. Indoxacarb, chlorantraniliprole, and lufenuron were Category 1, indicating they were harmless. Emamectin benzoate, chlorfenapyr, and bisultap were Category 2, indicating slightly to moderately toxic.

### 3.2. Sublethal Effects of Indoxacarb on T. howardi Development and Fecundity

#### 3.2.1. Percentage of Parasitism and Emergence

In general, the parasitism rate and emergence rate of the parasitoids decreased with the increase in indoxacarb concentration compared with those of the control (Table 2). The parasitism rate of adult exposure on the glass tube (R1) decreased by 26.6%, 35.2%, and 44.94% at sublethal concentrations of LC_20_, LC_30_, and LC_40_, respectively, compared with that of the control (*F* = 71.90; *p* = 0.0001; *df* = 3, 11), while adult exposure to pesticide on the host (R2) decreased by 11.5%, 20.34%, and 26.8%, respectively (*F* = 50.77; *p* = 0.0001; *df* = 3, 11). The parasitism rate of these two methods was significantly different under the same concentration. The parasitism rate of R2 was higher than R1 under the concentration of LC_40_ (*F* = 55.94; *p* = 0.0017; *df* = 1, 5). A similar trend was observed under the concentrations of LC_30_ (*F* = 50.76; *p* = 0.0021; *df* = 1, 5) and LC_20_ (*F* = 29.33; *p* = 0.0056; *df* = 1, 5). The lack of larval exposure to pesticides through host exposure (R3) data is because *T. howardi* was directly parasitized before being treated with the pesticide.

Under the concentrations of LC_20_, LC_30_, and LC_40_, the emergence rate of R1 decreased by 9.13%, 16.23%, and 23.36% (*F* = 28.76; *p* = 0.0001; *df* = 3, 11), R2 decreased by 5.64%, 11%, and 15.7% (*F* = 24.78; *p* = 0.0002; *df* = 3, 11), and R3 decreased by 9.5%, 15.66%, and 24.2% (*F* = 34.90; *p* = 0.0001; *df* = 3, 11), respectively, compared with that of the control (Table 2). No significant difference was found in the emergence rate under the three treatments at the same concentration. However, the emergence rate of R2 was the highest at the concentration of LC_40_ (*F* = 3.60; *p* = 0.0941; *df* = 2, 8) compared to the other two methods. A similar result was found with the concentrations of LC_30_ (*F* = 1.97; *p* = 0.2199; *df* = 2, 8) and LC_20_ (*F* = 2.43; *p* = 0.1688; *df* = 2, 8).

#### 3.2.2. Offspring Female Ratio 

**Figure 2:**
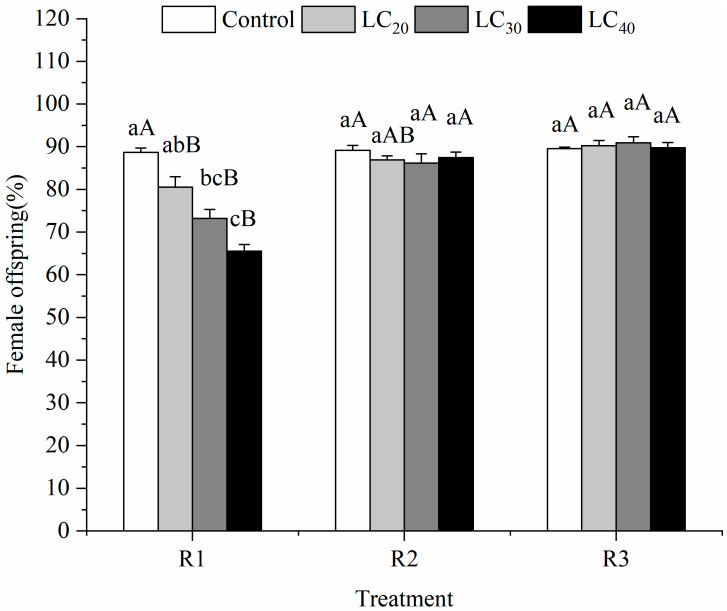
The offspring female ratio (mean ± SE) of *Tetrastichus howardi* at different concentrations of indoxacarb was treated with three methods. R1 (Routes of exposure 1) is adult exposure to pesticide residues on the glass tube, the LC_20_, LC_30_, LC_40_, and water control were used. R2 (Routes of exposure 2) is adult exposure to pesticide residues on the host, the LC_20_, LC_30_, LC_40_, and water control were used. R3 (Routes of exposure 3) is larval exposure to pesticides through host exposure, the LC_20_, LC_30_, LC_40_, and water control were used. Means in the same routes of exposure followed by different lowercase letters represent a significant difference in different concentrations of pesticide on *T*. *howardi.* Means in the same concentrations followed by the same uppercase letter were not significantly different in different routes of exposure based on Tukey’s multiple range test at 5% level.

The results showed that indoxacarb significantly decreased the female ratio of the progenies under the R1. (Figure 2). This ratio decreased with the increase in concentration, and the corresponding female ratio was the lowest at the concentration of LC_40_, which decreased by 23.14% compared with that of the control group (*F* = 28.14; *p* = 0.0001; *df* = 3, 11). However, no significant effect was observed on the ratio of offspring to females in the R2 and R3 of *T. howardi* treated with three concentrations of pesticides, with values between 86.13% and 89.13% (*F* = 0.77; *p* = 0.5415; *df* = 3, 11) and between 89.53% and 90.93% (*F* = 0.29; *p* = 0.8303; *df* = 3, 11), respectively. Therefore, when the concentration was LC_40_, the R1 method of exposure elicited the lowest female offspring ratio compared to the other two methods (*F* = 97.86; *p* = 0.0001; *df* = 2, 8). The concentrations of LC_30_ (*F* = 22.36; *p* = 0.0017; *df* = 2, 8) and LC_20_ (*F* = 8.63; *p* = 0.0172; *df* = 2, 8) mg L^−1^ showed the same results.

#### 3.2.3. Number of Emerged Offspring

**Figure 3:**
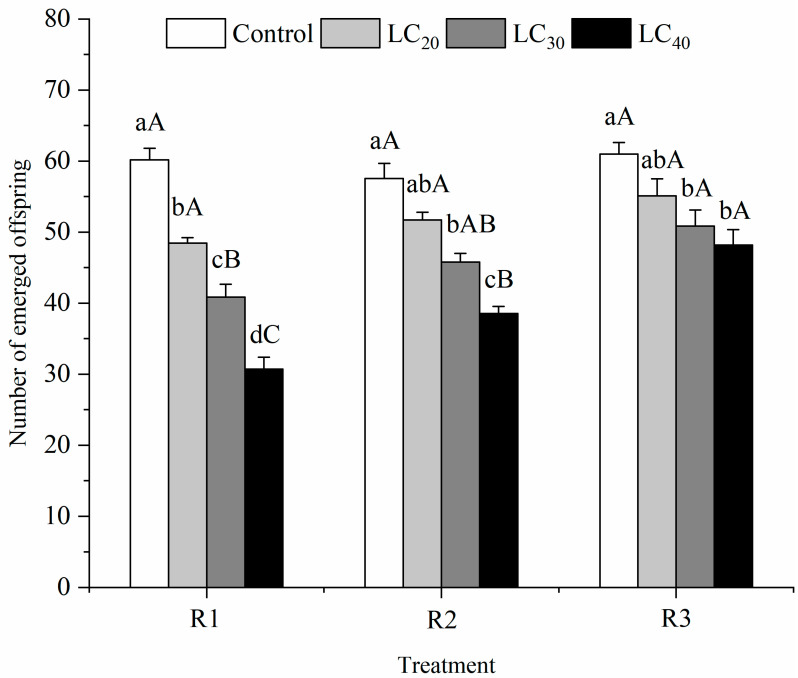
A number of emerged offspring (mean ± SE) of *Tetrastichus howardi* at different concentrations of indoxacarb were treated with three methods. R1 (Routes of exposure 1) is adult exposure to pesticide residues on the glass tube, the LC_20_, LC_30_, LC_40_, and water control were used. R2 (Routes of exposure 2) is adult exposure to pesticide residues on the host, the LC_20_, LC_30_, LC_40_, and water control were used. R3 (Routes of exposure 3) is larval exposure to pesticides through host exposure, the LC_20_, LC_30_, LC_40_, and water control were used. Means in the same routes of exposure followed by different lowercase letters represent a significant difference in different concentrations of pesticide on *T*. *howardi.* Means in the same concentrations followed by the same uppercase letter were not significantly different in different routes of exposure based on Tukey’s multiple range test at the 5% level.

The number of emerged offspring was inversely related to the pesticide concentration (Figure 3). Under R1, the number of emerged offspring was reduced from 60.17 to 30.73 (*F* = 66.10; *df* = 3, 11; *p* = 0.0001) as the concentration increased. Under R2, it decreased from 57.57 to 38.53 (*F* = 33.36; *df* = 3, 11; *p* = 0.0001), and under R3, it decreased from 60.97 down to 48.20 (*F* = 6.82; *df* = 3, 11; *p* = 0.0135) when *T. howardi* was present on the diet containing indoxacarb with three kinds of concentrations compared with the control group. Among the three concentrations, the number of emerged offspring reached the lowest when the concentration was LC_40_. Meanwhile, at the same sublethal concentration, the number of emerged offspring differed among the three methods (R1, R2, and R3). Statistically, when the concentration was LC_40_, the results showed R1 < R2 < R3 (*F* = 27.49; *p* = 0.0010; *df* = 2, 8). The same trend was observed at LC_30_ (*F* = 7.64; *p* = 0.0224; *df* = 2, 8) and LC_20_ (*F* = 4.44; *p* = 0.0656; *df* = 2, 8).

#### 3.2.4. Developmental Duration

**Figure 4:**
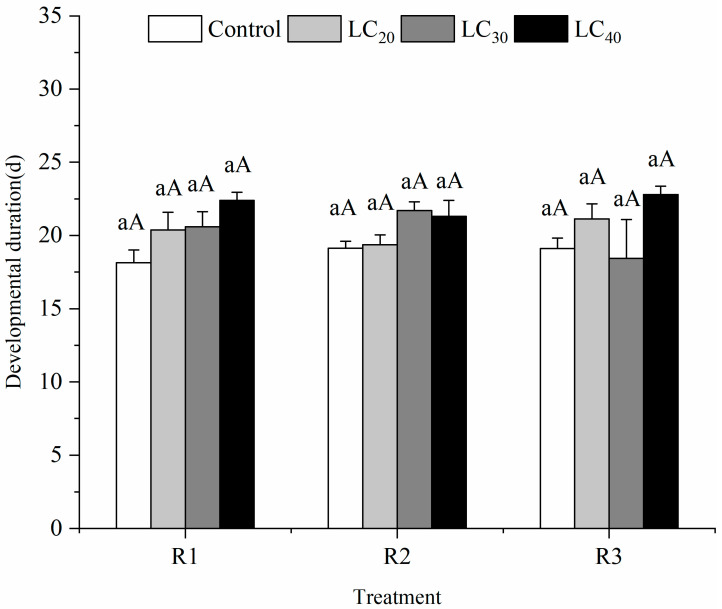
The development duration (mean ± SE) of *Tetrastichus howardi* at different concentrations of indoxacarb was treated with three methods. R1 (Routes of exposure 1) is adult exposure to pesticide residues on the glass tube, the LC_20_, LC_30_, LC_40_, and water control were used. R2 (Routes of exposure 2) is adult exposure to pesticide residues on the host, the LC_20_, LC_30_, LC_40_, and water control were used. R3 (Routes of exposure 3) is larval exposure to pesticides through host exposure, the LC_20_, LC_30_, LC_40_, and water control were used. Means in the same routes of exposure followed by different lowercase letters represent a significant difference in different concentrations of pesticide on *T*. *howardi.* Means in the same concentrations followed by the same uppercase letter were not significantly different in different routes of exposure based on Tukey’s multiple range test at the 5% level.

The developmental duration of R1 under the three concentrations was approximately 20.37–22.4 days (*F* = 3.39; *p* = 0.0745; *df* = 3, 11), that of R2 was roughly 19.37–21.7 days (*F* = 3.12; *p* = 0.0880; *df* = 3, 11), and that of R3 ranged from 18.43 days to 22.8 days (*F* = 1.77; *p* = 0.2303; *df* = 3, 11), respectively (Figure 4). Even though no significant difference was observed, the developmental duration of *T. howardi* treated by different concentrations of indoxacarb was prolonged compared with that of the control. At the LC_40_ concentration, no effect of the three methods (*F* = 0.10; *p* = 0.4230; *df* = 2, 8) on developmental duration was demonstrated in the experiment. A similar trend was observed in the concentration of LC_30_ (*F* = 0.98; *p* = 0.4269; *df* = 2, 8) and LC_20_ (*F* = 0.79; *p* = 0.4977; *df* = 2, 8).

## 4. Discussion

This study quantified the toxic effects of six insecticides on *T. howardi* and the adverse effects of indoxacarb on its development and fecundity. The toxicity of indoxacarb to parasitoid was relatively low among the six pesticides. This result is similar to Nozad-Bonab et al. who used different insecticides to *Trichogramma* *brassicae* [28]. In this research, indoxacarb showed the lowest risk quotient, with RQ = 7.43, whereas bisultap showed the highest, with RQ = 2250.00. This result indicated that indoxacarb is considered a relatively safe compound that is more friendly to natural enemies than other pesticides, according to the ratio between field recommended doses and the LC_50_ of the *T. howardi*. A similar outcome was obtained for *T. nubilale* (Hymenoptera: Trichogrammatidae) [29]. This finding may be connected to the unique mechanism of indoxacarb, which is a sodium-channel-blocking insecticide that blocks the flow of sodium ions to nerve cells, preventing the transmission of nerve impulses that could lead to paralysis and insect death [30,31]. Therefore, it is recommended to consider indoxacarb as a field insecticide to prevent and control *S. frugiperda*. However, even if the pesticide is relatively safe to natural enemies, the long-term exposure to insecticide sublethal environments can affect the life cycle of parasitic wasps.

For the lowest RQ of indoxacarb on parasitoid, how pesticides may act on parasitic wasps in a field environment was stimulated, and the effects of indoxacarb at different sublethal concentrations under three routes of exposure on the development and fecundity of *T. howardi* were measured. Compared with the control, the higher the concentration of pesticides, the more inhibited the propagation and development of parasitic wasps. Furthermore, under three methods, adult exposure to pesticide residues on the glass tube had the worst effect on development and reproduction. These results are similar to those of Souza et al., who revealed that the use of pesticides significantly inhibited the parasitism rate of *T. howardi* [32]. With the increase in the sublethal concentration of indoxacarb, the parasitism rate decreased gradually. Under the different methods, the parasitism rate of R1 was significantly lower than that of R2 at the same concentrations. Possibly because the pesticides directly applied to organisms before mating in R1, thus making the female wasps have an antifeedant effect and reduced energy intake, which affected the parasitic ability of the *T. howardi* and reduced the detection ability of the host. In R2, the pesticides were applied to pupae before parasitization. In such a case, the parasitism ability of *T. howardi* was relatively complete to be indirectly affected by pesticides. However, the parasitism rate under this method decreased significantly compared with the control group, possibly due to the influence of pesticide residues on the pupae [33].

The pesticide concentration was negatively related to the emergence rate and the number of emerged offspring. The results indicated that offspring development was under stress after pesticide treatment. The eggs laid by *T. howardi* were fed on the pupae treated by insecticides. The absorption of nutrients by the host was speculated to be inhibited, resulting in the reduction of the emergence rate and the number of emerged offspring [34]. The data in the present study showed that the emergence rate of R1 and R3 was lower than that of R2 at the same concentration. Under R1, the growth and development of the eggs were possibly prevented by the pesticide in the female wasps before they parasitized. Insecticides disrupt the coordination between the insect nervous system and hormonal system, leading to a breakdown in behaviors related to oviposition. Under R3, after the female wasps laid eggs, the pupae were impregnated with the pesticide. The offspring may be indirectly affected by the internal nutrition of the insecticide-treated pupae and directly affected by the exposure to pesticides during pupal immersion. The eggs were directly exposed to the pesticide, thus inhibiting the development of eggs and even directly dying and leading to the failure of emergence [35].

The effect of indoxacarb on the offspring female ratio of *T. howardi* only showed a significant difference in the treatment where adults were exposed to pesticide residues on the glass tube. In addition, the concentration of indoxacarb was negatively significantly correlated with the offspring female ratio in this method. The reason is because the Hymenoptera which produces female offspring requires the involvement of paternal genes to activate the expression of sex-determining genes in the maternal line for the fertilized egg to develop [36]. Under R2 and R3, the pupae were treated with pesticides after *T. howardi* mated, and the fertilized eggs were fully developed. Only in R1 was the *T. howardi* treated with pesticides before mating, and the sex determination mechanism may be influenced. Moreover, the carrier of sperm may be lost in the maternal line. The more significant the toxicity of indoxacarb is, the greater the carrier loss and the number of fertilized eggs decreased [37]. Therefore, the ratio of female offspring decreased.

The fertility and development of parasitoids are affected by insecticides [38], thus blocking the control effect on pests. Choosing a suitable pesticide that kills pests without causing too much damage to natural enemies is recognized as necessary.

## 5. Conclusions

This study elucidated that indoxacarb had relatively low toxicity to parasitic wasps and recommended spraying it in the field. Moreover, this study indicated that different exposure patterns and concentrations of pesticides have varying degrees of side effects on natural enemies. Even if the pesticide concentrations are low, such exposures can affect the fertility and development of natural enemies. We should strengthen the application methods to coordinate chemical and biological control in production. The stage of application of pesticides or measurement of seed coating should be considered to reduce the adverse effects of pesticides, thus minimizing the damage to natural enemies and improving the control efficiency. We expect to provide guidance for a more scientific and comprehensive application of pesticides and release natural enemies in the field. Furthermore, this experiment is significant for coordinating chemical and biological control of *S. frugiperda* in the field. However, all experiments were carried out in the laboratory and further field research needs to be conducted. 

## Figures and Tables

**Table 1:** Risk assessment of emamectin benzoate, chlorfenapyr, indoxacarb, chlorantraniliprole, bisultap, and lufenuron on *Tetrastichus howardi*.

Chemical Pesticide	LC_50_ (mg a.i L^−1^)	Regression Equation	χ^2^ Value	*p ^a^*	95% Confidence Limits	Recommended Dose (g a.i ha^−1^)	Risk Quotient (RQ)	Category
Emamectin benzoate	0.09	y = 2.140 + 2.065x	11.08	0.60	0.075~0.110	12	133.33	2
Chlorfenapyr	0.29	y = 1.117 + 0.900x	5.37	0.97	0.225~0.355	72	248.28	2
Indoxacarb	5.38	y = −1.292 + 0.768x	20.59	0.20	4.406~6.541	40	7.43	1
Chlorantraniliprole	0.84	y = 0.145 + 1.896x	11.01	0.61	0.663~1.034	30	35.71	1
Bisultap	0.30	y = 1.186 + 2.283x	8.10	0.84	0.243~0.366	675	2250.00	2
Lufenuron	0.91	y = 0.036 + 0.373x	10.53	0.65	0.457~1.867	45	49.45	1

RQ = Recommended dose (g a.i ha^−1^)/LC_50_ of *T. howardi.* Category = 1: harmless; 2: slightly to moderately toxic. ^a^ *p*-value associated with the chi-square, goodness-of-fit test.

**Table 2:** The parasitism rate (%) and emergence rate (%, mean ± SE) of *Tetrastichus howardi* at different concentrations of indoxacarb were treated with three methods.

Treatments	Parasitism Rate (%)			Emergence Rate (%)		
Concentration (mg L^−1^)	R1	R2	R3	R1	R2	R3
LC_40_	24.83 ± 1.76 cB	43.97 ± 1.77 cA	---------	65.77 ± 2.20 cA	72.67 ± 2.24 cA	64.03 ± 2.78 cA
LC_30_	34.57 ± 1.79 bB	50.43 ± 1.24 cA	---------	72.9 ± 2.20 bcA	77.37 ± 1.13 bcA	72.57 ± 2.28 bcA
LC_20_	43.17 ± 2.18 bB	59.27 ± 1.99 bA	---------	80.00 ± 1.93 bA	82.73 ± 0.90 bA	78.73 ± 0.84 bA
Control	69.77 ± 2.84 aA	70.77 ± 1.39 aA	---------	89.13 ± 1.24 aA	88.37 ± 1.04 aA	88.23 ± 0.76 aA

R1 (Routes of exposure 1) is adult exposure to pesticide residues on the glass tube, the LC_20_, LC_30_, LC_40,_ and water control were used. R2 (Routes of exposure 2) is adult exposure to pesticide residues on the host, the LC_20_, LC_30_, LC_40,_ and water control were used. R3 (Routes of exposure 3) is larval exposure to pesticides through host exposure, the LC_20_, LC_30_, LC_40,_ and water control were used. Means in the same columns followed by different lowercase letters represent a significant difference in different concentrations of pesticide on *T*. *howardi.* Means in the same row followed by the same uppercase letter were not significantly different in different routes of exposure based on Tukey’s multiple range test at 5% level.

## Data Availability

All data sets presented in this study are included in the article and can be made available by the authors upon reasonable request.
